# Supplementary material for: Development and application of species ID and insecticide resistance assays, for monitoring sand fly Leishmania vectors in the Mediterranean basin and in the Middle East
Source: PLoS Negl Trop Dis. 2024 Dec 3;18(12):e0012408. doi: 10.1371/journal.pntd.0012408 (PMC11642961; doi:10.1371/journal.pntd.0012408)
Supplement: S1 Fig — Authors’ personal photograph collection during experiments. Image " a " by Sofia Balaska, September 2022; Image " b " by Latifa Remadi, September 2022. (DOCX) [file pntd.0012408.s001.docx]

S1 Fig.

1.
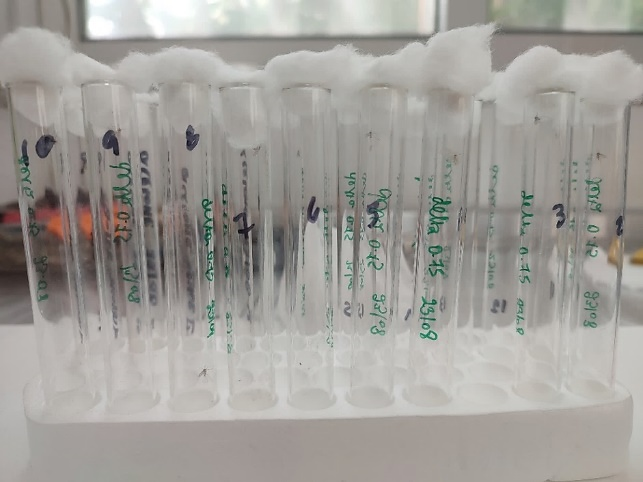

2.
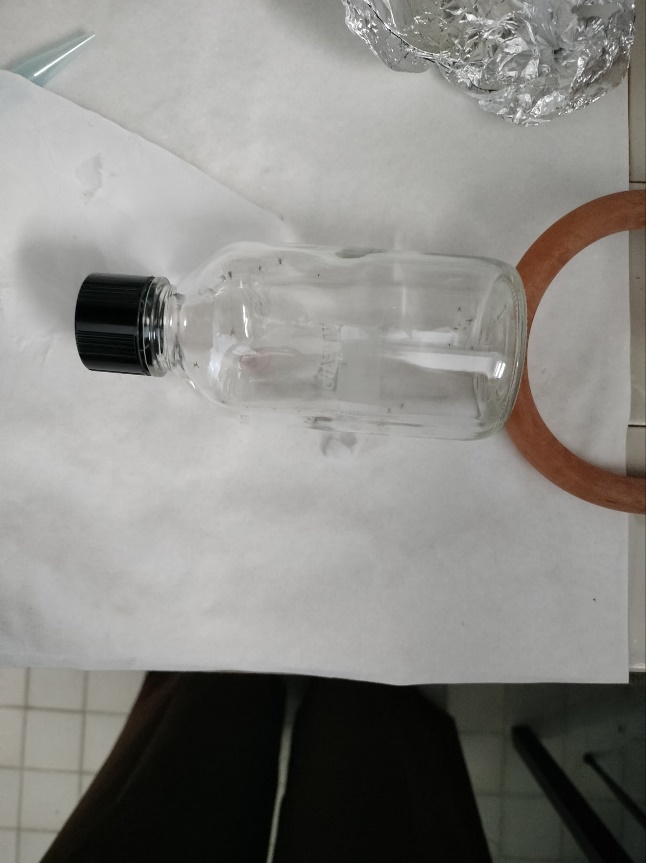


Sand fly bioassays against deltamethrin 0.75 μg/ml (a) in glass vials for individual exposure, compared to (b) standard CDC bottles used for grouped exposure.

(Authors’ personal photograph collection during experiments. Image a by Sofia Balaska, September 2022; Image b by Latifa Remadi, September 2022).
