# Supplementary material for: Development and application of species ID and insecticide resistance assays, for monitoring sand fly Leishmania vectors in the Mediterranean basin and in the Middle East
Source: PLoS Negl Trop Dis. 2024 Dec 3;18(12):e0012408. doi: 10.1371/journal.pntd.0012408 (PMC11642961; doi:10.1371/journal.pntd.0012408)
Supplement: Attachment — Submitted filename: Responses to reviewers comments.docx [file pntd.0012408.s003.docx]

**Dear Dr. Vontas, 
Thank you very much for submitting your manuscript "Development and application of species ID and insecticide resistance assays, for monitoring sand fly Leishmania vectors in the Mediterranean basin and in the Middle East" for consideration at PLOS Neglected Tropical Diseases. As with all papers reviewed by the journal, your manuscript was reviewed by members of the editorial board and by several independent reviewers. The reviewers appreciated the attention to an important topic. Based on the reviews, we are likely to accept this manuscript for publication, providing that you modify the manuscript according to the review recommendations.**

*We are thankful to the three reviewers and the Editor for carefully revising and accepting our manuscript, as well as for their valuable corrections and constructive recommendations. After considering all of the comments, we reworked on the manuscript and improved it, clarifying or correcting specific points in the text, tables and figures, as suggested. All of the reviewers’ comments are addressed point-by-point below. The line numbering in our responses corresponds to the revised clean version of the manuscript.*

**Reviewer #1: There is a dearth in entomological data for most vector-borne diseases and the authors aim to fill that gap and to develop tools that may assist others to do so. They use the term “macroarea” but this is beyond the scope of their work and suggest refining.**

*Thanks for your comment, we agree and changed “macroarea” to “area”, in line 40.*

**Objectives are ok, the text needs to be tightened. Ln 128 the data only enriches this small area within each country. State “our objectives are 1. Etc”**

*Thanks for your comment. We changed/ “toned down” this sentence (lines 129-131), which now reads “Our molecular and bioassay data enrich the scientific evidence for the IR status of distinct sand fly populations in areas of countries with overall poor data coverage, aiming to support evidence-based vector control efforts.”*

**Differing collection methods were used in sites, some comment should be made on possible impact. Why were these sites selected?**

*The parameters considered to select the sampling locations are described in detail in lines 136-140.* *Within each location, two or three distinct sites were selected based on technical/practical issues, i.e. presence of branches to hang CDC light traps, sites protected from direct exposure to light, surfaces where sand flies could rest, etc. Different collection methods were indeed used in different sites; for example, in Iran there was no availability of CDC light traps, so we switched to aspirating. We now point that possible impact in discussion lines 423-424.*

**A scale is needed on the map.**

*Thanks to all reviewers’ suggestions on Figure 1. The map is now changed/improved and adjusted to the journal’s requirements.*

**While data is collected on sites environment it is not really discussed anywhere in detail.**

*The selection of sampling sites and the description of the environment is now described in more detail in materials/methods lines 136-140, complementary to Table 1.*

**Why were CDC bottle assays not completed in other places? This is a weakness in the data that should be discussed.**

*Bioassays were not conducted in populations from Iran, Turkey and few locations in Greece (i.e. Rethymno and Chania), as low numbers of alive specimens were caught, occasionally due to unfavourable weather conditions. We understand that this might be a limitation in our study, however we did not have the ability to perform more consecutive night collections in these places. A sentence to discuss this limitation is now added in the discussion section, lines 462-466. Thank you for your suggestion.*

**Are the actual sand fly collections done inside homes? If so, was ethics obtained?**

*Sand fly collections were not conducted inside homes.*

**Reviewer #2: Methods used are suitable and the novel bioassay described here is very relevant for monitoring of insecticide resistance in sand flies.
Some further comments:
1. Sampling areas, sand fly collection and sample handling
- Table 1: replace (X, Y) with Coordinates**

*We corrected as suggested.*

**- Figure 1: could the top map, showing locations in Greece and Turkey, be slightly readjusted so location TU4 is now almost off the map, whereas there is no need to show so much of the sea in the eastern part.**

*Thanks to all reviewers’ suggestions on Figure 1. The map is now changed/ improved and adjusted to the journal’s requirements.*

**- Line 161: was obtained FROM… (not by)**

*We corrected as suggested, line 163.*

**- Are there any data on the number of human VL/CL cases in the areas where samples were collected?**

*Thanks for your comment. We included some relevant epidemiological data in the discussion lines 392-417, linked to the species structure.*

**5. Insecticide susceptibility bioassays
- It would be useful to provide a reason why the doses generally used for Aedes and Culex, rather than Anopheles, mosquitoes were used here.**

*Thanks for your question. The deltamethrin dose applied firstly in 2019 after samplings in Attica, Heraklion and Thessaloniki, Greece, was 10 ppm adapted from the CDC guidelines (https://www.cdc.gov/malaria/resources/pdf/fsp/ir_manual/Global-Insect-Resist-Bottle-Bioassay-508.pdf) as the diagnostic for Aedes mosquitoes, which was a bit lower than the one recommended for Anopheles (12.5 ppm). Later, CDC published new diagnostic doses (*[*https://www.cdc.gov/mosquitoes/pdfs/CONUS-508.pdf*](https://www.cdc.gov/mosquitoes/pdfs/CONUS-508.pdf)*) applying to Aedes and Culex species (0.75 ppm for deltamethrin), so we tested this just to stay consistent, since the one previously tested was too high to observe any possibly tolerant/resistant phenotype.*

**- Please provide number of females tested per bottle (even if it is a range).**

*The number of females exposed per bottle was 15-20 and is now mentioned in the respective materials & methods section, in line 213-217. We also added in brackets the total number of specimens subjected to bioassays per population, shown in figures 2 and 3.*

**Section ii on individual glass vial bioassays:
- It would be good to provide a picture of this novel method (especially if this novel bioassay could be pictures next to a standard CDC bottle).**

*Thanks for the suggestion. We included a picture of the deltamethrin coated glass vials vs. the regular CDC bottle bioassays as supplementary figure 1.*

**- Were the flies not kept for 24 hours to record mortality? If so, why not? Surely this would make it more comparable with the standard CDC bottle bioassay described in the previous section.**

*Generally, the standard exposure to deltamethrin was for 1 hour. Less than 20% of the specimens (identified lately to their vast majority as P. simici) displayed “tolerance” within this first hour of exposure, and were exposed for 2 hours totally. Mortality was recorded after 24 hours of recovery. We now clarified the experimental process in both materials & methods and results section, lines 246-253.*

**Reviewer #1: The species diversity is well presented – with different vectors in Greece compared to Turkey/Iran. Is there any link to disease burden that can be made with this?**

*Thanks for your comment. We tried to “link” the species distribution with some epidemiological data of each respective region, in discussion lines 392-417, by adding some extra information. However, we prefer not to draw further conclusions/ associations, as in some cases (e.g. locations from Turkey) the sample size is very small.*

**KDR is an interesting presentation, but here all species have grouped into one it would be good for places like Adana that individual species are shown. Although it is assumed the appendix is broken down into granular forms?**

*Although in table 3, mutant samples are indeed grouped together for simplicity, in the respective results section 2 (lines 286-304), we mention in detail all the identified species harbouring kdr mutations in all sampling locations. All granular data are now provided in the Supporting Information file.*

**Unfortunately, bioassays were only done in Greece that had no real evidence of kdr, (would have been good to compare to Adana). While significantly reducing the deltamethrin concentration used in the assay, reduced the knockdown rate, that would be expected**.

**The individual assay is a good way to separate out species differences. How easy this could be applied in a vector control programme should be discussed.**

*This methodology is indeed challenging to be part of vector control programs, a point that we already mentioned in the discussion section. We now extended the sentence/comment in lines 488-491, “Although we perceive that…regular CDC bottle bioassays***”**.

**It was good to see that species ID of specimens were done, but it may have been interesting to include individual kdr here as well.**

*All specimens exposed to deltamethrin in our study, either CDC or individual bioassays, were genotyped individually for kdr. Samples from Thessaloniki subjected to individual bioassays in 2022, are grouped with those collected in 2019 (Table 3), as no kdr mutation was recorded.*

**Reviewer #2: Results are clearly presented and described.**
**Some further comments:
- Table 2: please add codes of sampling sites to their names (e.g. GR1 next to Attica/Kalentzi). Or add in the footnote which sites GR2-GR3 and TU2-TU3 were.**

*Thanks for the comment, we added the respective regions’ names in the footnote for clarity.*

**- Line 274: Heterozygosity in P. Bobbi specimen is mentioned. Please provide both codons - or did you mean this was a 1014F/S heterozygote?**

*Thanks for noticing that. We mean 1014L/1014F heterozygosity in P. tobbi (line 292-293) and the genotype is now clarified in line (TTA/TTT). The heterozygote 1014F/S was identified as P. papatasi (line 294-295).*

**- Line 281: heterozygosity in Iranian P. papatasi - please provide the codons observed.**

*The identified genotype for these heterozygote P. papatasi samples is now mentioned clearly in line 301.*

- **Figures 2 and 3: It should be “Knock down rate following deltamethrin exposure…” and not “…upon Deltamethrin…”.**

*Thanks for noticing that, we corrected both figures as suggested.*

**- Were sand flies that were used in bioassays (either CDC or novel individual ones) then also genotyped? It would make sense to provide results giving both phenotype and genotype data as both are necessary to assess the insecticide resistance status - mutations by themselves, even if associated with resistance, do not necessarily mean the specimens are resistant. For example, in India it is known that P. argentipes are resistant to DDT, have kdr mutations, yet still appear susceptible to pyrethroids. Provision of phenotypic and genotypic data is crucial for surveillance and control efforts, especially since leishmaniasis control largely depends on insecticides/vector control.**

*Thanks for your constructive comment. Yes, all sand flies subjected to bioassays (CDC or individual) were afterwards identified to species and genotyped for kdr, and this is now clarified in lines 226 and 252. We would have definitely provided any association between molecular and phenotypic data, however bioassays were performed only against the populations originating from Attica, Heraklion and Thessaloniki, Greece, in which mutations did not occur.* *We totally agree with your comment on the association between kdr and resistance, thus we added that as a discussion point in lines 456-460, as well.*

**Reviewer #1: Overall, the conclusions are supported by the data, but in some instances this may need to be toned down a little. However, this is a public health issue globally and surveillance in this area needs to increase not just for sand flies but all vector-borne diseases.
Ln 396 – 397 recall over time can be problematic this should be mentioned.**

*Thanks for noticing that, we deleted the sentence from the discussion section.*

**401-404 – Bangladesh, and India have reported high kdr levels as referenced  - however what has not been discussed is that these populations still remain susceptible to pyrethroids in bioassay that are currently in use for sand fly control and effective VL elimination (DOI: 10.1016/S1473-3099(24)00420-1) the low impact of kdr alone has also been observed in malaria transmitting mosquitoes. While similar correlations cannot be made with this data due to the lack of bioassay data combined with kdr it needs to be discussed here.**

*We thank reviewer #1 for this comment. We agree to that, as stated also above in reviewer’s #2 similar comment. Populations from Turkey with high kdr frequencies in our study were not defined as “pyrethroid resistant” at any point of our manuscript, due to absence of bioassay data to support. We rather refer to kdr-positive sand flies result as a possible indicator of resistance/tolerance to pyrethroids. To make this clear and avoid any misunderstanding, we are now discussing that in lines 465-466 and added some relevant references [51 and 52], to meet both reviewers’ suggestions.*

**417 – totally agree, and as the manuscript mentions the WHO sandfly assays it would be good to mention if those are higher, lower or same as the mosquito ones previously used. It is a good indicator as to how far off the current assays may be.**

*Thanks for this valuable comment, we compared the WHO mosquito vs. sand fly diagnostic doses and indeed there are significant differences; DC for sand fly species might be higher or lower than the ones established for mosquitoes, in an insecticide-dependent manner. We now discuss this point in lines 468-473.* **The individual bioassays are an interesting concept – although operationally in a programme may be challenging. Have any sample size calculations been attempted on the number sand flies needed to collect, test to get a viable data set?**

*We discuss the difficulty in applying this protocol in routine vector control campaigns in lines* *488-491. At least 20-30 individuals per species should be needed to get some statistical significance, however we did not address this valuable direction in this study and the links with operational impact.*

**Reviewer #2: Discussion and Conclusions are clear and supported by the data presented.

Some further comments:
- IR profiling tools are mentioned. Having a tool similar to IR Mapper for malaria vectors would be hugely beneficial for all those involved in leishmaniasis research, surveillance and control efforts. I am not suggesting that the authors should make such a tool but it would be good to see something like it mentioned in Discussion.**

*Thanks for your suggestion. That’s an interesting point, so we incorporated that in the last sentence of the discussion, where we already mention the malaria paradigm. The sentence in lines 501-505 now reads “Following the paradigm of mosquito surveillance, for which the existing IR profiling/ reporting databases (e.g. IR Mapper) have greatly supported decision making, developing similar tools for sand flies and tailoring Leishmania infection surveillance systems to IVM would benefit sustainable leishmaniasis control activities”.*

**- Paragraph starting in line 381: these differences are indeed interesting. Surely there is a need for further research on bionomics of these sand fly species and populations (and others) to inform the control and surveillance efforts.
- Paragraph in lines 393-404: yes, regional IR surveillance is definitely needed, providing both phenotypic and genotypic data. But another crucial thing is lack of good control tools - and the fact that we clearly need to stop relying on the use of pyrethroids and start using other insecticides suitable for public health purposes.**

*Thanks for your valuable comment. Following your suggestion, we now discuss some points on the current leishmaniasis chemical control strategies in this paragraph, lines 447-452, “Such findings highlight…insecticide application regime lately”.*

**- Line 429: if starting a sentence with a species name, spell the genus name out in full.**

*Thank you, we corrected as suggested.*

**- In line with what is suggested in Conclusions (lines 465-468): To further improve both species identification, insecticide resistance genotyping and detection of pathogens, an approach based on high-throughput barcoding/amplicon seq might also be beneficial, especially as morphological identification of sand flies is much more complicated than that of mosquitoes.**

*Thank you for your comment. According to your suggestion, we modified the sentence in lines 523-525, which now reads “Last but not least, there is an urgent need to generate relevant genomic resources for sand flies, and develop high-throughput methodologies to characterise IR mechanisms, that would also be beneficial for the identification of novel resistance diagnostic markers”.*

**Reviewer #1: It would be good to see kdr data where it exists presented by species as this is a consistent drive by the paper.**

*Although in table 3, mutant samples are grouped together for simplicity, in the respective results section 2, we mention in detail all the identified species harbouring kdr mutations in all sampling locations, as well as their genotypes.*

**The map could be clearer with appropriate scales etc.**

*Thanks to all reviewers’ suggestions on Figure 1. The map is now changed/ improved and adjusted to the journal’s requirements.*

**Assume granular data will be available in appendices**

*Yes, all granular data are now provided in a Supporting Information file.*

**Reviewer #2: Overall: language should be slightly revised, some sentences are awkward.**

*Thank you for your comment. The manuscript is carefully revised and the language is improved where needed.*

**Reviewer #3: The article by Balaska et al reports interesting findings in a not well investigated field, insecticide resistance in sand flies. Moreover the paper proposes a protocol for testing in single sand flies, to overcome the impossibility to easily define sand fly species. In my opinion this is a relevant work, I have only minor revision to propose.**

**62 specify the geographic area, worldwide?**

*Yes, this refers to the mortality recorded globally. We corrected accordingly, line 64.*

**67 71 sentence not clear, please revise**

*We revised the sentence, which now reads “Some key determinants accounting for this changing epidemiological trend are the climatic crisis along with environmental modifications/urbanization, facilitating the spread of sand flies northwards, and occasionally in urban settings, as well as the increasing population movement (i.e. migration/ mass population displacement, tourism, travelling with reservoir hosts, such as dogs)”, in lines 69-73.*

**174 please clarify if pcr was performed in single on every specimen**

*Yes, specimens were analyzed individually for species ID and kdr mutations, as clarified now in lines 179 and 189.*

**241 please specify the maximum time of the observation**

*Thank you, we now describe the bioassay process more clearly, in lines 249-250.*

**213 reported in materials and methods**

*Sorry, this comment is not very clear to us. Does the reviewer refer to the sentence in lines 216-217 “Alive and knocked-down sand flies in each bottle were recorded at time intervals of 5–15 minutes, for 1 hour exposure time.”? And if yes, what’s to correct/change?*

**215 217 this sentence seems a materials and methods part**

*Lines 215-217 of the initially submitted manuscript are already part of the materials & methods.*

**246 I suggest to insert more details on molecular identifications, as percentages of identity with deposed sequences and/or a tree showing obtained clusters.**

*More details on the molecular identification of species are now added in materials/method 176-177 and results lines 257-258.*

**table 2 I suggest to report also the number of sand flies and percentages between brackets**

*Thank you for your suggestion, we changed table 2 and included the number of samples identified per species in each population.*

**333 the number of sand flies tested is unclear, please specify**

*The total number of specimens (N) analyzed phenotypically here is mentioned in figure 4, but we also added the numbers in the relative results section, in lines 370-372*.


**Reviewer #1: The paper delivers more data to the entomological surveillance community which is much needed. It highlights some of the critical gaps that need filing to make informed decisions on fighting vector-borne diseases.
While it would have been nice to see a correlation made between kdr and bioassay data, it is good to see that Greece has yet to develop a kdr issue.**

**Please use place names in text and delete all codes, G1, G2 etc for a) consistency and b) readability**

*We deleted almost all location codes throughout the manuscript, as recommended, except for some few points connected to a respective figure (e.g. Fig2 and Fig3 and relative text in the results section).*

**As colony sand flies not used, wild caught can be dropped**

*Thanks for your suggestion, we deleted “wild-caught”, as well as replaced “wild populations” with “populations”, where necessary.*

**18 remove “upcoming”**

*“Upcoming” is now deleted.*

**44-46- rephrase**

*Thanks for the suggestion, we simplified the sentence: “Phlebotomine sand flies transmit leishmaniasis to human and animals, a neglected tropical disease of the (sub-)tropics, currently expanding though in previously non-endemic regions”, lines 44-45.*

**49-key are capacity and resources**

*Thanks for your comment. We agree that capacity building and human resources are key factors hindering research on sand flies IR, therefore changes the sentence in lines 47-50, which now reads “IR data are limited in sand fly populations worldwide though, due to the unavailability of robust molecular diagnostic and phenotyping tools, as well as deficiencies in human resources, appropriate training and capacity building.”*

**57 – how?**
*Thanks you. We revised the sentence, which now reads: “Our study contributes data for sustainable evidence-based sand fly control, and highlights the importance of systematic IR surveillance programs in leishmaniasis endemic regions.”, in lines 57-59.*

**71-possible**

*The sentence in lines 69-73 is now rephrased, upon other reviewer’s suggestion.*

**88- suggest “WHO recently established”**

*We corrected as suggested, line 91.*

**107-108 – change to “hinder” rather than “deter”**

*We corrected as suggested, line 109.*

**120 – replace “include” – “are”**

*We replaced as suggested, line 122.*

**249-251- not easy to read**

*Thank you. The whole paragraph is now rephrased, in lines 259-277.*

**358 – care as reads 3 countries per region**

*In this sentence, “three...” is directly followed by “…endemic countries”, which are mentioned at the end. However, to avoid any misunderstanding we deleted “three” (line 383-387).*

**357 – replace wild – “sand fly”**

*We deleted “wild” as suggested, line 385.*

**359-delete on-focus**

*It is now deleted, as suggested, line 387.*

**414-does the sand fly population live in pyrethroid sprayed environment?**

*Exactly, population from the American Farm School comes from a habitat regularly sprayed with pyrethroids, as mentioned in table 1.*

**450 – “significantly” is a stretch.**

*Thanks for your suggestion, we deleted “significantly”.*
